# Infantile Status Epilepticus: A Case of Excessive Water Intake in a Five-Month-Old Girl

**DOI:** 10.7759/cureus.59115

**Published:** 2024-04-26

**Authors:** Abeer Alshaikh, Abdulrahman A Aldoseri, Raafat Hammad Seroor Jadah

**Affiliations:** 1 Pediatrics, Bahrain Defense Force Hospital, Riffa, BHR; 2 Pediatrics and Child Health, Bahrain Defense Force Hospital, Riffa, BHR

**Keywords:** seizure, water intoxication, status epilepticus, hyponatremia, pediatrics

## Abstract

Hyponatremia in children, especially in normal infants below the age of six months, is a common cause of the first onset of afebrile convulsions, which can be rarely associated with water intoxication and can lead to a state of encephalopathy and status epilepticus if not diagnosed and managed properly early. Water intoxication is an uncommon but potentially lethal cause of hyponatremia. We report a five-month-old girl who presented to our hospital with status epilepticus, facial puffiness, cyanosis, and severe hyponatremia secondary to water intoxication. Proper investigations and labs were done, and the patient was managed successfully. The aim of reporting this case is to highlight the importance of water intoxication with secondary status epilepticus in infants below six months of age.

## Introduction

Status epilepticus secondary to water intoxication is a rare condition in infancy, which is characterized by somnolence or irritability, followed by seizures, and is associated with hyponatremia [1]. Water intoxication typically manifests clinically as nausea, vomiting, altered mental status, edema, hypothermia, and seizures [2]. Pathophysiologically speaking, slow correction at a rate of less than 0.5 mmol/L/hour is preferable in both acute and chronic hyponatremia. A rapid correction of severe hyponatremia can lead to complications in the brain, especially in the central pontine and extrapontine myelinolysis [1,3,4]. The prognosis has varied in the cases that have been described, from total recovery to permanent debilitation [2].

Infantile status epilepticus is a medical emergency that necessitates immediate evaluation and intervention. It is crucial to consider underlying conditions that may cause seizures, particularly when clinical features are atypical. Water intoxication can lead to seizures in infants, and this case report elaborates on the presentation, diagnosis, and effective management of a child with status epilepticus resulting from water intoxication.

## Case presentation

A five-month-old girl without medical history presented to our emergency department with jerky movements of her left limbs, which had been ongoing for approximately 25 minutes. The symptoms had commenced shortly after bottle-feeding, accompanied by facial cyanosis, frothing, and upper limb stiffness. Family members also noted facial and body puffiness.

The infant had been receiving frequent formula feedings every one to two hours, supplemented with 100 mL of water every three hours in between feedings, and had recently initiated the weaning process with solid foods. The father reported polyuria, with a diaper change occurring every two to three hours. The infant was delivered at full term with no postnatal complications, although there was a positive family history of seizure disorders in a second-degree relative.

Upon physical examination, the patient exhibited normal vital signs and temperature, although she appeared initially drowsy with eyelid puffiness and intermittent eye-opening. Abdominal examination revealed distension. Initial laboratory investigations revealed unremarkable complete blood count, renal function test, and liver function test but with severe hyponatremia (108.5 mmol/L, normal range: 135-145 mmol/L).

**Figure 1:**
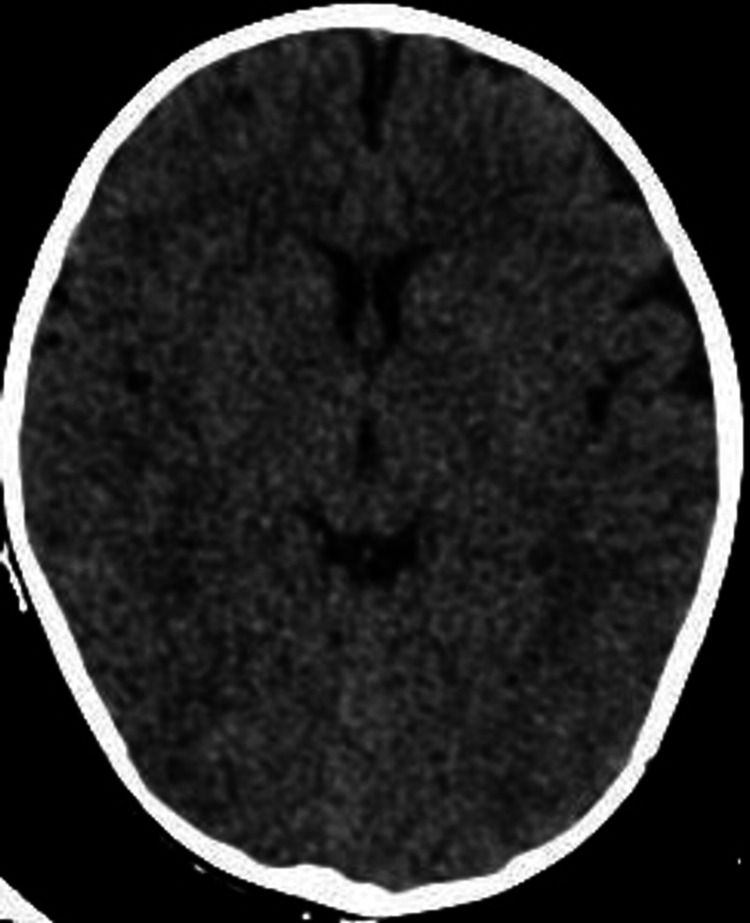
Axial view of the brain CT scan showing normal anatomical findings. CT: computed tomography

**Figure 2:**
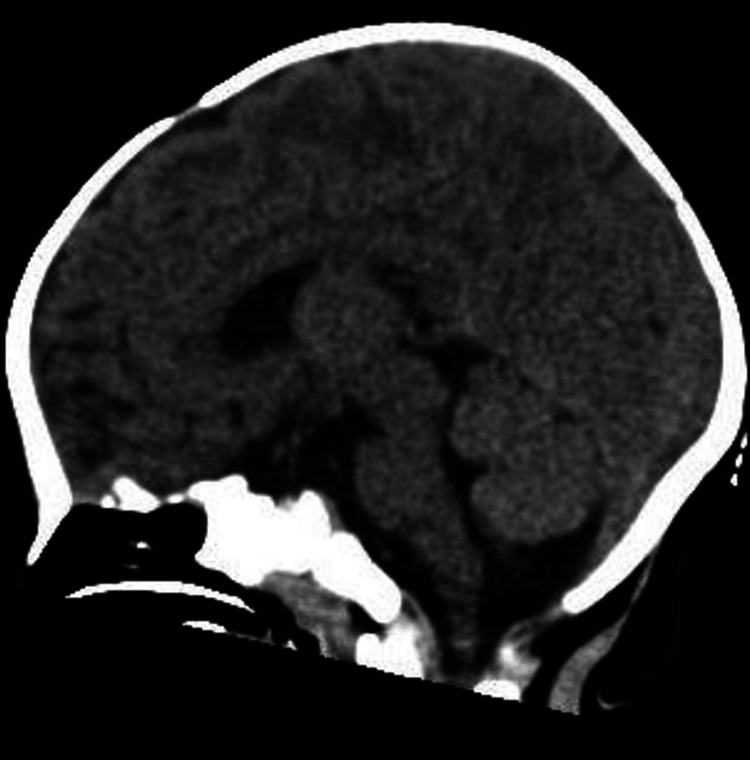
Sagittal view of the brain CT scan displaying no pathological findings.

The initial management included the administration of 3% sodium chloride (NaCl) at 3 mL/kg (18 mL) over 20 minutes, rectal diazepam, and intravenous diazepam to control the patient's seizures. A second seizure episode was successfully treated with Keppra 20 mg/kg and then 10 mg/kg. Subsequent sodium levels improved to 133.5 mmol/L. Initial brain computed tomography (CT) scan showed no remarkable findings (Figures 1, 2).

After successful management of the status epilepticus, a neurological examination revealed a normal infant. Tone and power examinations were within normal limits, deep tendon reflexes were normal, and there were no signs of cerebellar dysfunction. Cranial nerve examination was intact, and no neurocutaneous skin lesions were observed. Other systemic examinations were unremarkable; parental counseling was done, which included proper feeding practice with proper fluids intake; the patient was discharged home with a complete recovery of clinical symptoms; and follow-up as an outpatient was done.

## Discussion

Hyponatremia is the main factor in new-onset non-febrile seizures in infants younger than six months of age with a normal exam; this mostly occurs as a result of improper feeding practices that result in water intoxication [2]. In this case, the infant had been receiving an excessive amount of water in between frequent formula feedings, which led to hyponatremia. Thus, it occurs when there is an excessive intake of water, which dilutes the body's sodium levels to dangerous lows. In infants, this condition can be triggered by various factors, including well-intentioned but excessive fluid administration, the use of diluted infant formula, or an overzealous approach to maintaining hydration.

The central challenge in managing infants with water intoxication is recognizing the condition. Symptoms can mimic other causes of seizures and encephalopathy, making it imperative to have a high index of suspicion, particularly in the presence of unusual clinical features. These features may include facial puffiness, as seen in this case, which can be attributed to the cerebral edema that occurs as a result of hyponatremia. Typical clinical presentation of water intoxication includes nausea, vomiting, altered mental status, edema, hypothermia, and seizures [2,5]. Like in our case, most hyponatremia cases caused by water intoxication show no abnormalities in brain CT [6].

As our patient was managed properly, seizure management included anticonvulsant medications with phenobarbital, diazepam, and fluid therapy ranging from 0.2% saline up to 3% saline. The time required to attain a serum sodium level greater than 130 mEg/L was less than 12 hours in all cases [1]. Fluid restriction and hypertonic saline infusion will allow for prompt resolution of the central nervous system manifestation and correction of electrolyte disturbances [3,6]. Fortunately, our patient was discharged home after fully recovering from clinical symptoms. Similar to the cases that have been reported, the prognosis has ranged from full recovery to lifelong disability. Most likely, prolonged seizures, hypoxia, and consequences of the sodium imbalance directly on the central nervous system will result in more severe outcomes [2].

This case underscores the critical importance of recognizing water intoxication and establishes the significance of nutritional history for figuring out a child's hyponatremia as a major contributor to non-febrile seizures in infants, and the key to preventing recurrence of water intoxication in infants is education and awareness [5,6].

## Conclusions

Infantile status epilepticus should prompt a comprehensive evaluation for underlying systemic issues, with water intoxication being a significant consideration. Effective management of water intoxication through sodium correction is essential in resolving seizures and strict parent education on proper feeding protocol especially in the infant age group is crucial to avoid the recurrence of water intoxication. This case report emphasizes the need to recognize and address water intoxication as a leading cause of seizures in infants, particularly when associated with atypical clinical features.

Parents and caregivers need to be informed about the potential risks associated with excessive fluid intake and dilution of infant formula. It is crucial to emphasize the importance of following recommended feeding guidelines and recognizing the signs of overhydration.
